# CD133 in brain tumor: the prognostic factor

**DOI:** 10.18632/oncotarget.14406

**Published:** 2016-12-31

**Authors:** Bin Li, Cian M. McCrudden, Hiu Fung Yuen, Xinping Xi, Peng Lyu, Kwok Wah Chan, Shu Dong Zhang, Hang Fai Kwok

**Affiliations:** ^1^ Faculty of Health Sciences, University of Macau, Avenida de Universidade, Taipa, Macau SAR; ^2^ School of Pharmacy, Queen's University Belfast, Belfast, United Kingdom; ^3^ Institute of Molecular and Cell Biology, A*STAR, Singapore; ^4^ Department of Pathology, University of Hong Kong, Hong Kong; ^5^ Northern Ireland Centre for Stratified Medicine, Biomedical Sciences Research Institute, University of Ulster, Londonderry, United Kingdom

**Keywords:** brain tumor, CD133, HOX, LIM2, stem cell

## Abstract

CD133 has been shown to be an important stem cell factor that promotes glioma progression. However, the mechanism for CD133-mediated glioma progression has yet to be fully elucidated. In this study, we found that CD133 mRNA expression was a prognostic marker in three independent glioma patient cohorts, corroborating a putative role for CD133 in glioma progression. Importantly, we found that CD133 expression in glioma was highly correlated with the expression of HOX gene stem cell factors (HOXA5, HOXA7, HOXA10, HOXC4 and HOXC6). The expression of these HOX genes individually was significantly associated with survival. Interestingly, the prognostic significance of CD133 was dependent on the expression level of HOX genes, and vice versa. CD133 (*p* = 0.021) and HOXA7 (*p* = 0.001) were independent prognostic markers when the three glioma patient cohorts were combined (*n* = 231). Our results suggest that HOX genes may play a more important role in progression of glioma when CD133 expression is low. Furthermore, we showed that low-level expression of LIM2 in CD133-high glioma was associated with poorer survival, suggesting that LIM2 could be a therapeutic target for glioma expressing a high level of CD133. Connectivity mapping identified vinblastine and vincristine as agents that could reverse the CD133/HOX genes/LIM2-signature, and we confirmed this by *in vitro* analysis in glioma cell lines, demonstrating that CD133 and HOX genes were co-expressed and could be downregulated by vincristine. In conclusion, our data show that CD133 and HOX genes are important prognostic markers in glioma and shed light on possible treatment strategies for glioma expressing a high level of CD133.

## INTRODUCTION

Glioma is the most common type of primary brain tumor. Of the gliomas, grade III astrocytoma and grade IV glioblastoma are highly malignant, and afford patients a very poor prognosis, with only around 10% surviving for more than five years [1, 2]. Increasing evidence indicates that stem cell signaling plays an important role in the development of gliomas, thereby presenting a potential therapeutic target for their treatment [3]. Most importantly, glioma stem cells (GSCs) have been shown to be resistant to conventional therapies, leading to high treatment failure and recurrence in patients with unresectable glioblastoma [4]. Several genes and pathways have been linked to GSCs, such as Notch, bone morphogenetic proteins, NF-kB, Wnt, epidermal growth factor, and Shh [4].

Another Two GSC-linked genes are CD133 and nestin; CD133, also known as Prominin-1, is a glycoprotein that is encoded by the *PROM1* gene. Glioblastoma multiforme cells grown in neurosphere culture were CD133- and nestin-positive and capable of tumor formation *in vivo*, while 2-D cultured counterparts lacked expression and malignancy when intracranially implanted in nude mice [5]. CD133 plays an integral role in cancer progression by maintenance of stem cell properties of cancer cells, such as their ability to self-renew [6]. CD133 activates the Akt pathway to increase cellular resistance to chemotherapy [7], while it also promotes cancer cell self-renewal and growth through JNK signaling [8]. Prognostic values of CD133 expression have been shown in lung cancer [9], gastric cancer [10], colon cancer [11], esophageal cancer [12], thyroid cancer [13], pancreatic cancer [14] and ovarian cancer [15].

Almost a decade ago, a correlation between CD133-positivity in glioma specimens and poor patient prognosis was reported [16], and its prognostic significance in glioma has since been demonstrated in two independent meta-analyses [17, 18]. Molecular mechanisms of CD133-mediated glioma progression have been further revealed recently. CD133 overexpression inhibited chemotherapy-induced apoptosis in glioma cell lines *in vitro* [19, 20]. CD133-mediated temozolomide resistance in glioma cells was shown to be dependent on the activation of Notch and Shh pathways [21], while CD133 was shown to confer resistance to multiple chemotherapeutic agents via upregulation of CD90, CD44, nestin, Msi1 and MELK [20], and the Akt and NF-kB pathways [22]. The self-renewing property of CD133-positive cells was dependent on the activity of Akt [23, 24] and Notch pathways [25], and was enhanced in a hypoxic environment via HIF-1a [26]. SirT1, which plays an important role in governing the radiosensitivity of glioma cells, is a downstream target of CD133 [27].

The HOX gene family consists of highly conserved hoemodomain transcription factors that, when overexpressed, promote glioma initiating potential, poor prognosis and chemotherapeutic drug resistance [28]. Epigenetic alterations resulted in aberrant expression of HOX genes at its transcriptional level [28]. LIM2, lens intrinsic membrane protein 2, has been shown to maintain cytoskeletal integrity, cell morphology and intercellular communication in mouse lenses [29], but little was known about the role of LIM2 in cancer or in glioma.

In this study, we aim to identify genes that are co-expressed with CD133 in human glioma specimens, and to investigate how these co-expressed genes interact with CD133 and influence its prognostic value. Lastly, we aim to identify small molecules that could reverse the expression of CD133 and its associated genes.

## RESULTS

### The association between CD133 expression and survival of patients with glioma

**Figure 1:**
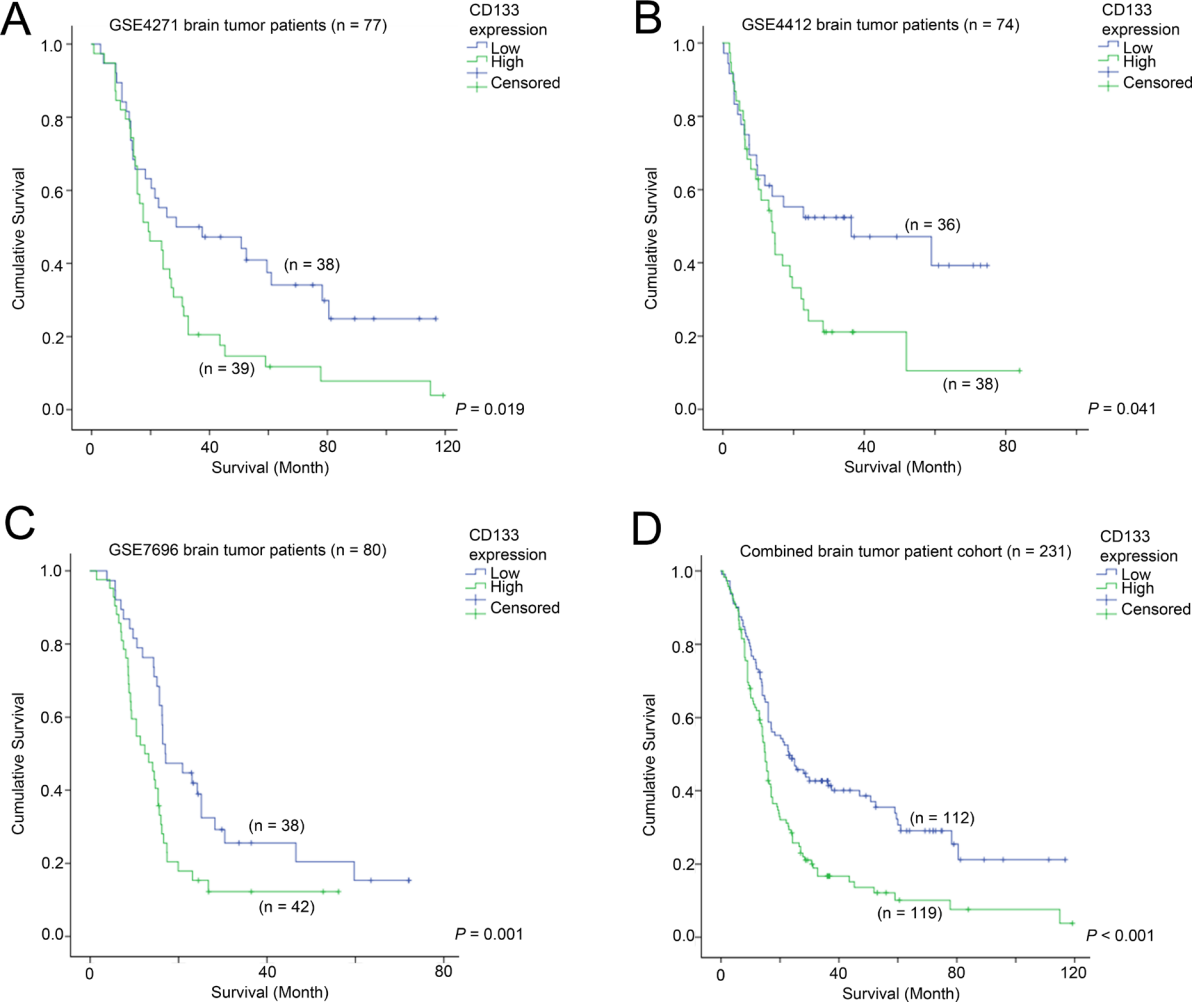
The association between CD133 and patient survival Kaplan-Meier analyses for CD133 mRNA expression in (**A**) GSE4271, (**B**) GSE4412, (**C**) GSE7696, and (**D**) the combined cohort.

The association between CD133 expression and survival of patients was tested in the three glioma patient cohorts with survival data available. In GSE4271 (*n* = 77; Figure 1A), GSE4412 (*n* = 74; Figure 1B) and GSE7696 (*n* = 80; Figure 1C), a high level expression of CD133 in the brain tumor was significantly associated with shorter survival. Similar results were obtained using Cox-regression analysis (Table 1); patients whose brain tumors expressed a higher level of CD133 had a significantly higher risk of progression or death in GSE4271, GSE4412 and GSE7696. When the three cohorts were combined, high-level expression of CD133 was significantly associated with a shorter survival time (hazard ratio of 1.81, a 95% confidence interval of 1.33 – 2.45 and a *p-value* of < 0.001). Patients whose tumors expressed a high level of CD133 had a mean survival time of 25.7 months compared to 45.7 months for patients whose tumors expressed a low-level of CD133 (Figure 1D; *p* < 0.001). The association between high level of CD133 and shorter survival was observed in patients regardless of whether temozolomide was added to radiotherapy as shown in Supplementary Figure 1.

**Table 1:** Cox regression analysis for CD133 mRNA in brain tumor cohorts

	GSE 4271			GSE 4412			GSE 7696	
HR	95% CI	*P*-value	HR	95% CI	*P*-value	HR	95% CI	*P*-value
1.23	1.01–1.50	0.037	1.285	1.00–1.66	0.053	1.34	1.08–1.66	0.007

### The association between CD133 expression and tumor grade

**Figure 2:**
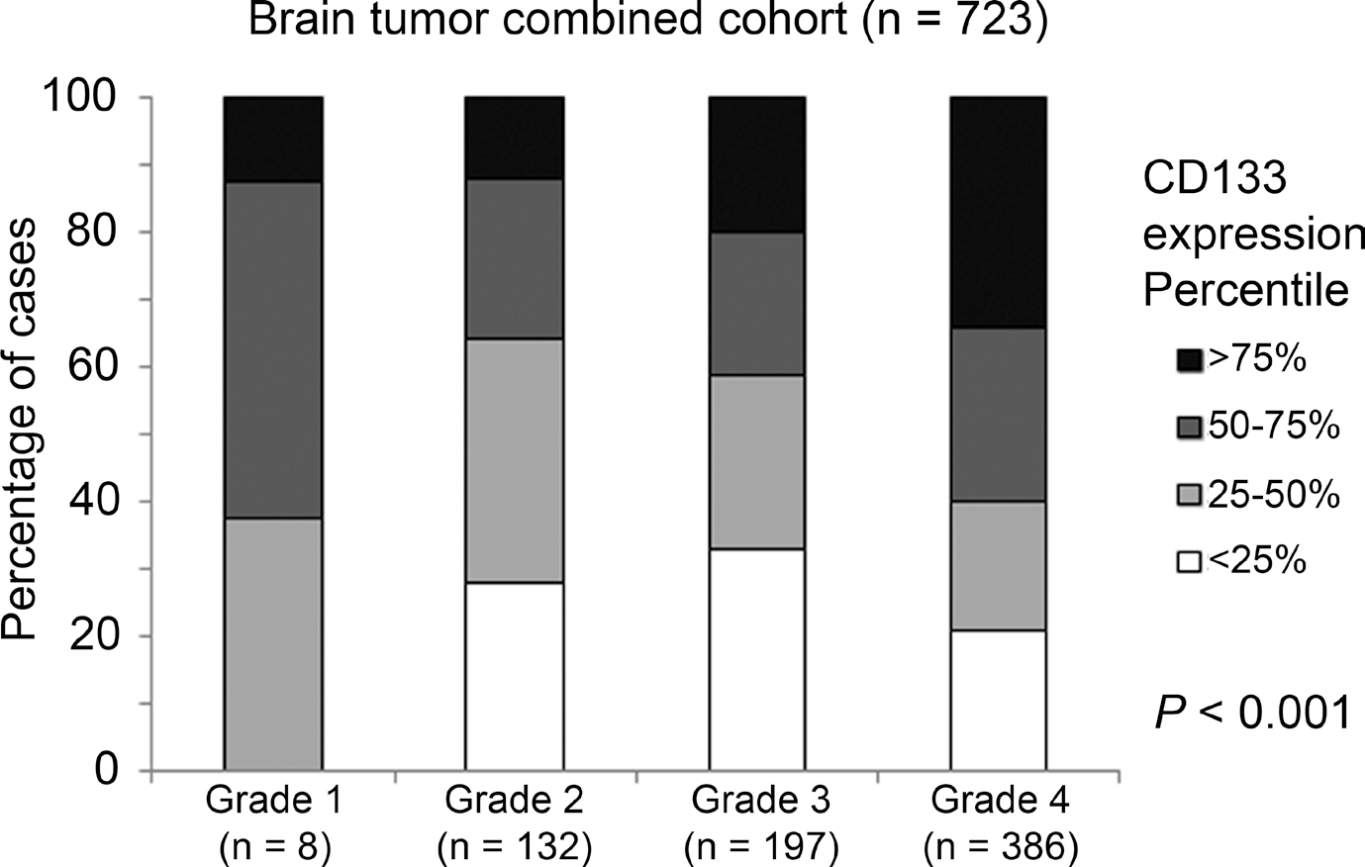
The association between CD133 mRNA expression and tumor grade A histogram displaying the association between CD133 mRNA expression profile and tumor grading in the combined cohort.

With the exception of GSE7696, all other five brain tumor patient datasets included in the current study provided information on tumor grade. The association between CD133 and tumor grade was tested in the combined cohort (with only five cohorts, omitting GSE7696) by Chi-Square analysis, and we found a significant correlation between CD133 expression and tumor grade (Figure 2; *p* < 0.001).

### Identification of CD133 co-expressed genes in glioma patient datasets

Upon analysis of probeset expression patterns in three independent brain tumor patient cohorts with survival data available (GSE4271, GSE4412 and GSE7696) stratified into CD133-high and –low subgroups, we found altered expression of several genes from the HOX family to be common to all three cohorts. HOXA5, HOXA7, HOXA10, HOXC4 and HOXC6 were significantly differentially expressed among CD133-high and CD133-low tumors in all three independent patient cohorts, and were selected for further study. As shown in Tables 2A, and 2C, expression of CD133 and the five HOX family genes were significantly correlated in GSE4271, GSE4412 and GSE7696, respectively.

**Table 2A:** GSE4271 brain tumor dataset (n = 100); correlation of CD133 and HOX gene expressions

			CD133	HOXA5	HOXA7	HOXA10	HOXC4	HOXC6
Spearman's rho test	CD133	Correlation Coefficient	1.000	.381^*^	.388^*^	.356^*^	.377^*^	.399^*^
		*P* value	.	< 0.001	< 0.001	< 0.001	< 0.001	< 0.001
	HOXA5	Correlation Coefficient	.381^*^	1.000	.787^*^	.769^*^	.544^*^	.682^*^
		*P* value	< 0.001	.	< 0.001	< 0.001	< 0.001	< 0.001
	HOXA7	Correlation Coefficient	.388^*^	.787^*^	1.000	.711^*^	.509^*^	.592^*^
		*P* value	< 0.001	< 0.001	.	< 0.001	< 0.001	< 0.001
	HOXA10	Correlation Coefficient	.356^*^	.769^*^	.711^*^	1.000	.515^*^	.586^*^
		*P* value	< 0.001	< 0.001	< 0.001	.	< 0.001	< 0.001
	HOXC4	Correlation Coefficient	.377^*^	.544^*^	.509^*^	.515^*^	1.000	.788^*^
		*P* value	< 0.001	< 0.001	< 0.001	< 0.001	.	< 0.001
	HOXC6	Correlation Coefficient	.339^*^	.682^*^	.592^*^	.586^*^	.788^*^	1.000
		*P* value.	< 0.001	< 0.001	< 0.001	< 0.001	< 0.001	.

**Correlation is significant at the 0.01 level (2-tailed).

*Correlation is significant at the 0.05 level (2-tailed).

**Table 2B:** GSE4412 brain tumor dataset (n = 85); correlation of CD133 and HOX gene expressions

			CD133	HOXA5	HOXA7	HOXA10	HOXC4	HOXC6
Spearman's rho test	CD133	Correlation Coefficient	1.000	.232*	.368^*^	.222*	.345^*^	.348^*^
		*P* value	.	= 0.032	< 0.001	= 0.041	= 0.001	= 0.001
	HOXA5	Correlation Coefficient	.232*	1.000	.658^*^	.636^*^	.495^*^	.552^*^
		*P* value	= 0.032	.	< 0.001	< 0.001	< 0.001	< 0.001
	HOXA7	Correlation Coefficient	.368^*^	.658^*^	1.000	.682^*^	.512^*^	.460^*^
		*P* value	< 0.001	< 0.001	.	< 0.001	< 0.001	< 0.001
	HOXA10	Correlation Coefficient	.222*	.636^*^	.682^*^	1.000	.671^*^	.544^*^
		*P* value	= 0.041	< 0.001	< 0.001	.	< 0.001	< 0.001
	HOXC4	Correlation Coefficient	.345^*^	.495^*^	.512^*^	.671^*^	1.000	.652^*^
		*P* value	= 0.001	< 0.001	< 0.001	< 0.001	.	< 0.001
	HOXC6	Correlation Coefficient	.348^*^	.552^*^	.460^*^	.544^*^	.652^*^	1.000
		*P* value.	= 0.001	< 0.001	< 0.001	< 0.001	< 0.001	.

**Correlation is significant at the 0.01 level (2-tailed).

*Correlation is significant at the 0.05 level (2-tailed).

**Table 2C:** GSE7696 brain tumor dataset (n = 84); correlation of CD133 and HOX gene expressions

			CD133	HOXA5	HOXA7	HOXA10	HOXC4	HOXC6
Spearman's rho test	CD133	Correlation Coefficient	1.000	.398*	.410^*^	.280^*^	.308^*^	.337^*^
		*P* value	.	= 0.032	< 0.001	= 0.01	= 0.004	= 0.002
	HOXA5	Correlation Coefficient	.398*	1.000	.710^*^	.694^*^	.582^*^	.660^*^
		*P* value	= 0.032	.	< 0.001	< 0.001	< 0.001	< 0.001
	HOXA7	Correlation Coefficient	.410^*^	.710^*^	1.000	.773^*^	.492^*^	.518^*^
		*P* value	< 0.001	< 0.001	.	< 0.001	< 0.001	< 0.001
	HOXA10	Correlation Coefficient	.280^*^	.694^*^	.773^*^	1.000	.535^*^	.590^*^
		*P* value	= 0.01	< 0.001	< 0.001	.	< 0.001	< 0.001
	HOXC4	Correlation Coefficient	.308^*^	.582^*^	.492^*^	.535^*^	1.000	.623^*^
		*P* value	= 0.004	< 0.001	< 0.001	< 0.001	.	< 0.001
	HOXC6	Correlation Coefficient	.337^*^	.660^*^	.518^*^	.590^*^	.623^*^	1.000
		*P* value.	= 0.002	< 0.001	< 0.001	< 0.001	< 0.001	.

**Correlation is significant at the 0.01 level (2-tailed).

*Correlation is significant at the 0.05 level (2-tailed).

### The association between the expression of HOX genes and survival of patients with glioma

HOX genes have been shown to play an important role in brain malignancies, with multiple HOX genes being found to be overexpressed in glioblastoma multiforme, the most aggressive glial tumor of the brain [30]. HOXA9 and HOXA10 genes were associated with a shorter survival in pediatric high-grade glioma patient samples [31], while elevated HOXA9 and HOXA10 gene methylation was associated with an increased survival probability [32].

**Figure 3:**
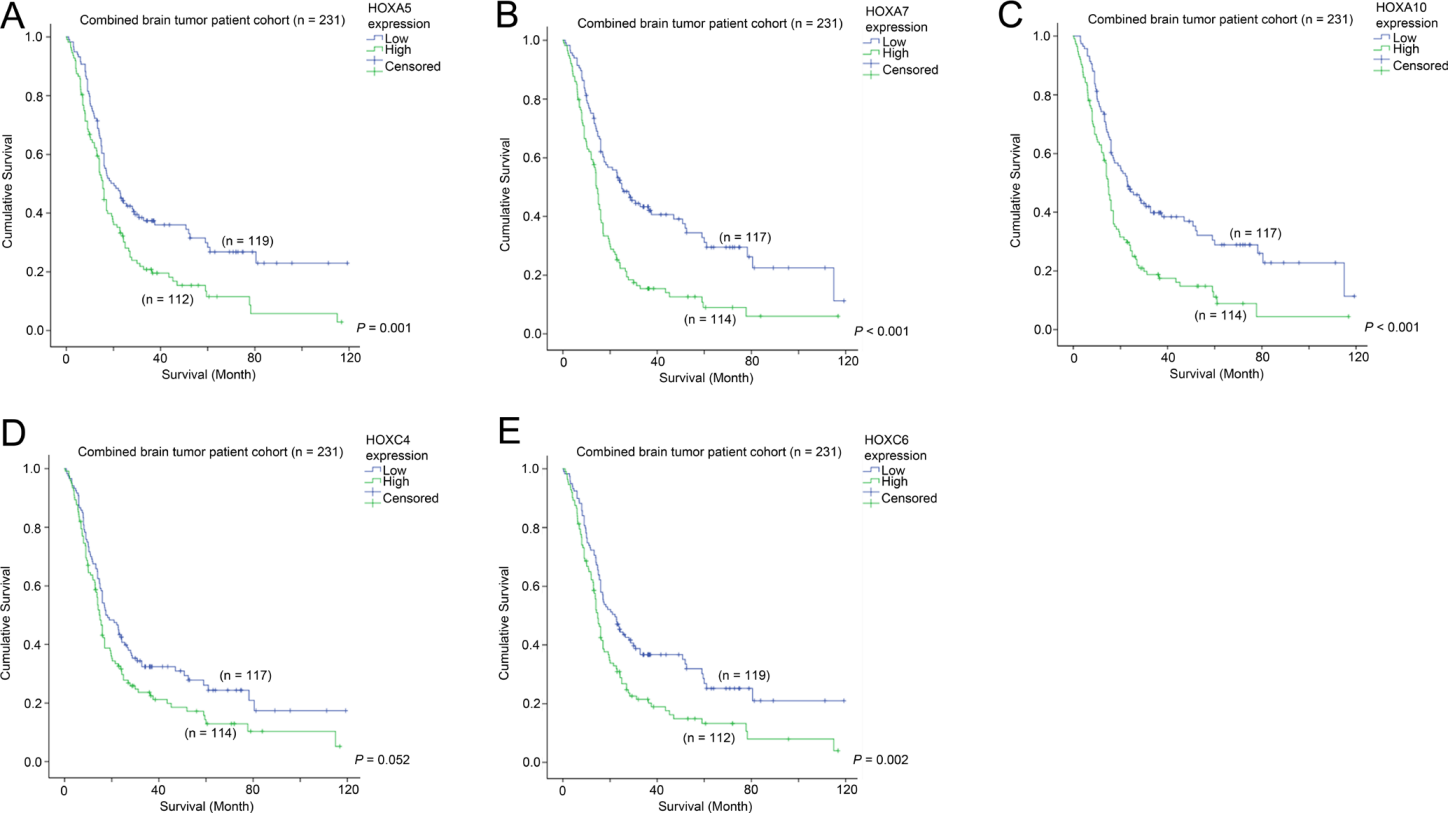
The association between mRNA expression of HOX genes and survival Kaplan-Meier analyses for (**A**) HOXA5, (**B**) HOXA7, (**C**) HOXA10, (**D**) HOXC4 and (**E**) HOXC6 mRNA expression in the combined cohort.

We went on to investigate whether the five commonly differentially expressed HOX genes were prognostic factors in the combined patient cohort for which survival data was available. As shown in Figure 3A, patients whose tumors expressed a high level of HOXA5 had a mean survival time of 26.3 months, compared to 44.9 months for those patients whose tumors expressed a low level of HOXA5 (Figure 3A; *p* = 0.001). Similarly, elevated HOXA7 (23.7 vs 47.2 months; Figure 3B; *p* < 0.001), HOXA10 (24.1 vs 46.1 months; Figure 3C; *p* < 0.001), HOXC4 (30.4 vs 41.0 months; Figure 3D; *p* = 0.052) and HOXC6 (27.1 vs 44.1 months; Figure 3E; *p* = 0.002) were associated with shorter patient survival. These results suggest that the five HOX genes identified to be co-expressed with CD133 are also prognostic factors in brain tumor patients.

### The prognostic significance of CD133 in patients with differential expression of HOX genes

Having determined that CD133 expression is highly correlated with the expression of HOX genes, and that HOX genes themselves confer prognostic significance in brain tumor patients, we went on to further investigate the prognostic value of CD133 in brain tumors expressing different levels of HOX genes. The mean survival time and 95% CI for different patterns of expression for different genes are listed in Supplementary Table 2.

**Figure 4:**
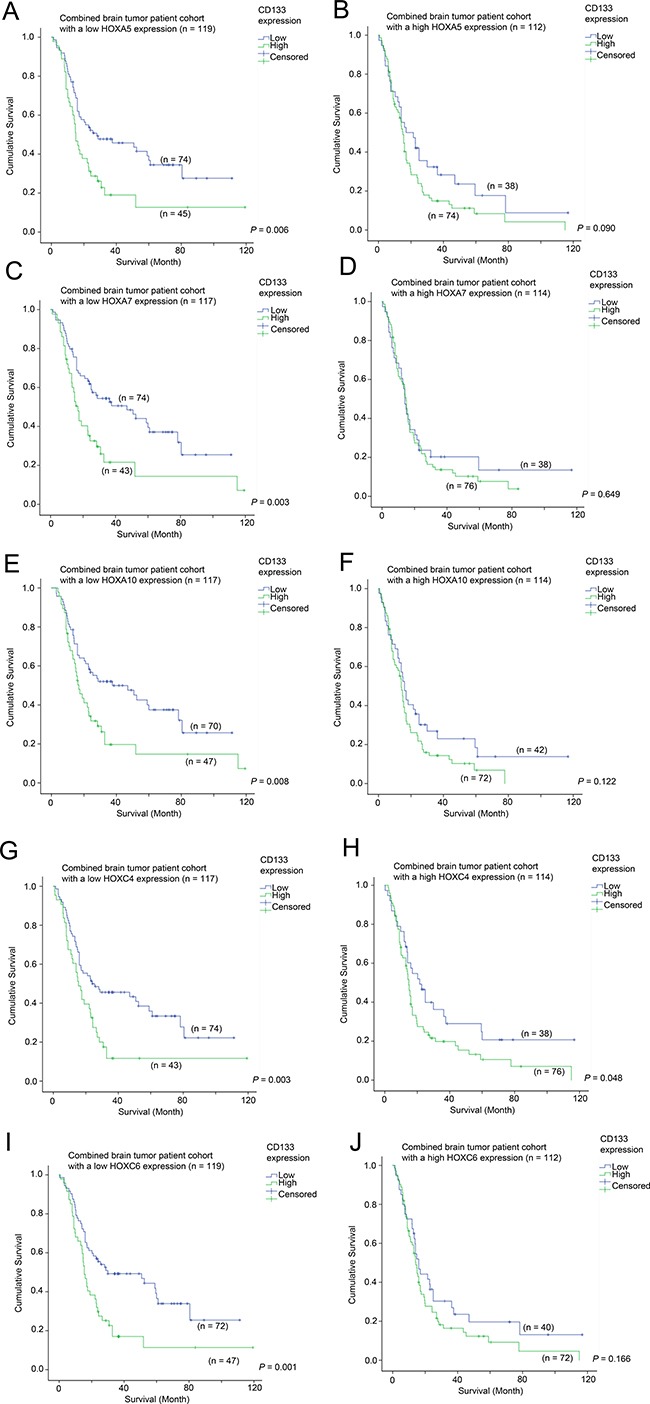
The association between CD133 mRNA expression and survival in glioma patients with different level of expression of HOX genes Kaplan-Meier analyses for CD133 mRNA expression in the combined cohort of glioma patients expressing (**A**) a low level of HOXA5, (**B**) a high level of HOXA5, (**C**) a low level of HOXA7, (**D**) a high level of HOXA7, (**E**) a low level of HOXA10, (**F**) a high level of HOXA10, (**G**) a low level of HOXC4, (**H**) a high level of HOXC4, (**I**) a low level of HOXC6, and (**J**) a high level of HOXC6.

Elevated expression of CD133 was significantly associated with a shorter survival in patients whose tumors expressed HOXA5 at a low level (30.3 vs 50.6 month mean survival; Figure 4A; *p* = 0.006), but there was no association between CD133 expression and survival in patients whose tumors expressed high levels of HOXA5 (Figure 4B; *p* = 0.090). Likewise, CD133 overexpression was significantly associated with shorter survival when HOXA7 (32.4 vs 53.0 months; Figure 4C; *p* = 0.003), HOXA10 (32.7 vs 52.2 months; Figure 4E; *p* = 0.008), HOXC4 (27.6 vs 47.4 months; Figure 4G; *p* = 0.003) and HOXC6 (29.0 vs 51.0 months; Figure 4I; *p* = 0.001) were lowly expressed. This association was not significant or only barely significant when HOXA7 (Figure 4D; *p* = 0.649), HOXA10 (Figure 4F; *p* = 0.122), HOXC4 (Figure 4H; *p* = 0.048) or HOXC6 (Figure 4J; *p* = 0.166) were themselves also overexpressed. The results were highly consistent among all the HOX genes identified in this study, suggesting that CD133 and HOX genes affect the aggressiveness of cancer via similar mechanisms.

### The prognostic significance of HOX genes in patients with differential expression of CD133

**Figure 5:**
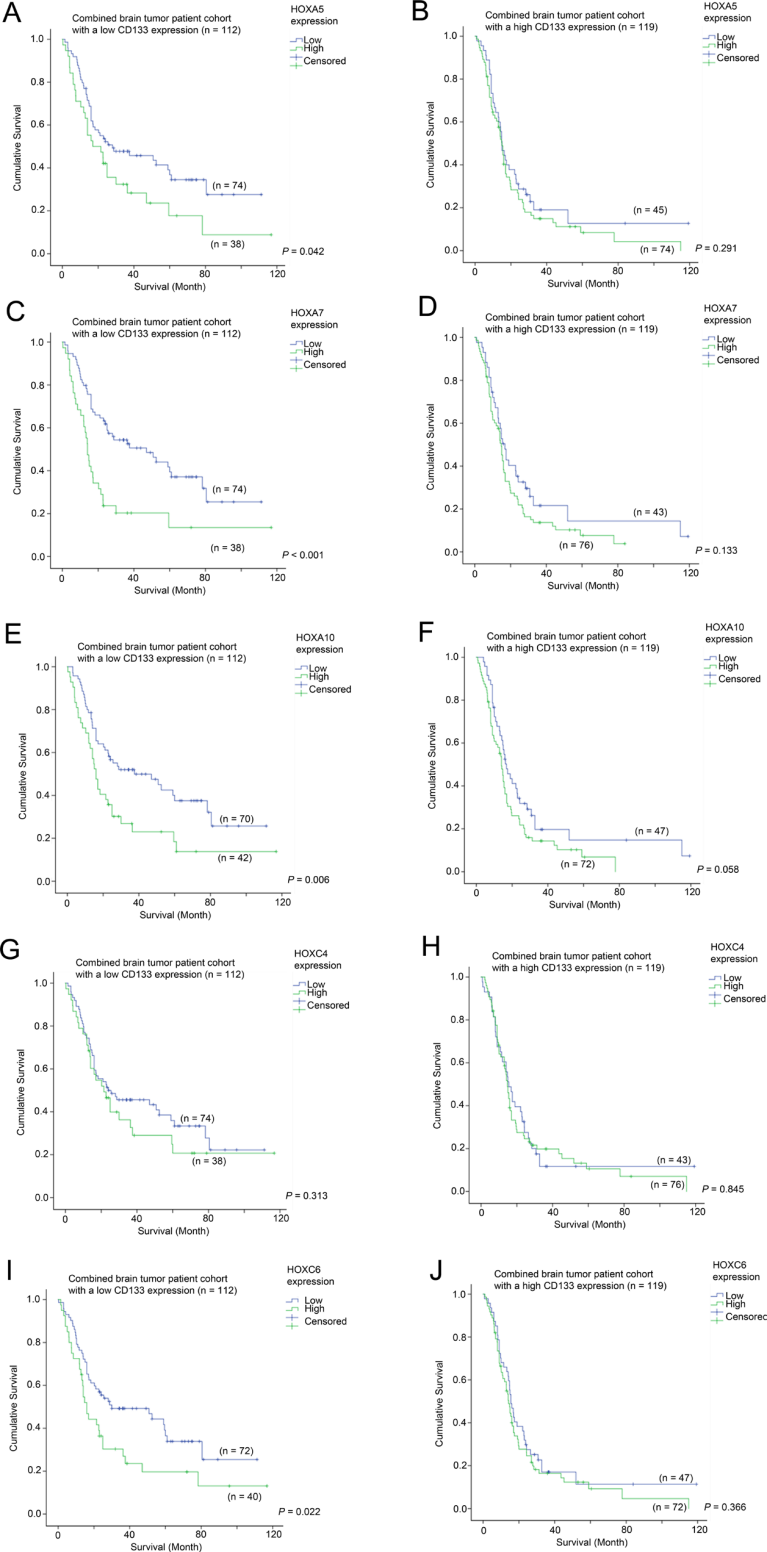
The association between mRNA expression of HOX genes and survival in glioma patients with different level of expression of CD133 Kaplan-Meier analyses for HOXA5 expression in (**A**) CD133-low and (**B**) CD133-high glioma, for HOXA7 expression in (**C**) CD133-low and (**D**) CD133-high glioma, for HOXA10 expression in (**E**) CD133-low and (**F**) CD133-high glioma, for HOXC4 expression in (**G**) CD133-low and (**H**) CD133-high glioma, and for HOXC6 expression in (**I**) CD133-low and (**J**) CD133-high glioma in the combined cohort.

Having demonstrated that HOX genes had prognostic potential in brain tumor patients, we went on to analyze whether the prognostic significance of HOX genes was affected by CD133 expression. Here, we present the data from Figure 4 in a different configuration in Figure 5 to illustrate the prognostic significance of HOX genes in specimens with different CD133 expression levels. Overexpression of HOX5A was predictive of a shorter survival in the CD133-low subgroup (33.1 vs 50.6 months mean survival; Figure 5A; *p* = 0.042), but not in the CD133 overexpressing subgroup (Figure 5B; *p* = 0.291). HOXA7 was predictive of survival in CD133-low (29.4 vs. 53.0 months; Figure 5C; *p* < 0.001), but not in CD133-high tumors (Figure 5D; *p* = 0.133). HOXA10 was similarly predictive of survival in CD133-low (32.2 vs. 52.2 months; Figure 5E; *p* = 0.006), but not in CD133-high tumors (Figure 5F; *p* = 0.058), as was HOXC6 predictive of survival in CD133-low (33.4 vs 51.0 months; Figure 5I; *p* = 0.022), but not in CD133-high tumors (Figure 5J; *p* = 0.366). Interestingly, HOXC4 expression was not predictive of survival in either CD133-low (Figure 5G; *p* = 0.313) or CD133-high (Figure 5H; *p* = 0.845) subgroups of patients. These results suggest that HOX genes may play a more important role in enhancing the aggressiveness of brain tumors with a low level expression of CD133.

### CD133 and HOXA7 genes are independent prognostic factors in brain malignancies

Multivariate Cox regression was performed to investigate whether CD133 expression and the expression of HOX genes identified in this study are independent prognostic factors. As shown in Table 3, by a forward stepwise approach with an entry limit of *p* < 0.05, only CD133 expression (*p* = 0.021) and HOXA7 expression (*p* = 0.001) were independent prognostic markers in brain tumor patients.

**Table 3:** Multivariate Cox-regression analysis for HOX genes and CD133

Factor	Hazard Ratio	95% Confidence Interval	*P*-value for inclusion
**CD133**	**1.467**	**1.059–2.032**	**0.021**
**HOXA5**	NS	NS	0.748
**HOXA7**	**1.767**	**1.277–2.446**	**0.001**
**HOXA10**	NS	NS	0.155
**HOXC4**	NS	NS	0.526
**HOXC6**	NS	NS	0.315

### Identification of genes that predict survival in patients with tumors expressing a high level of CD133

**Figure 6:**
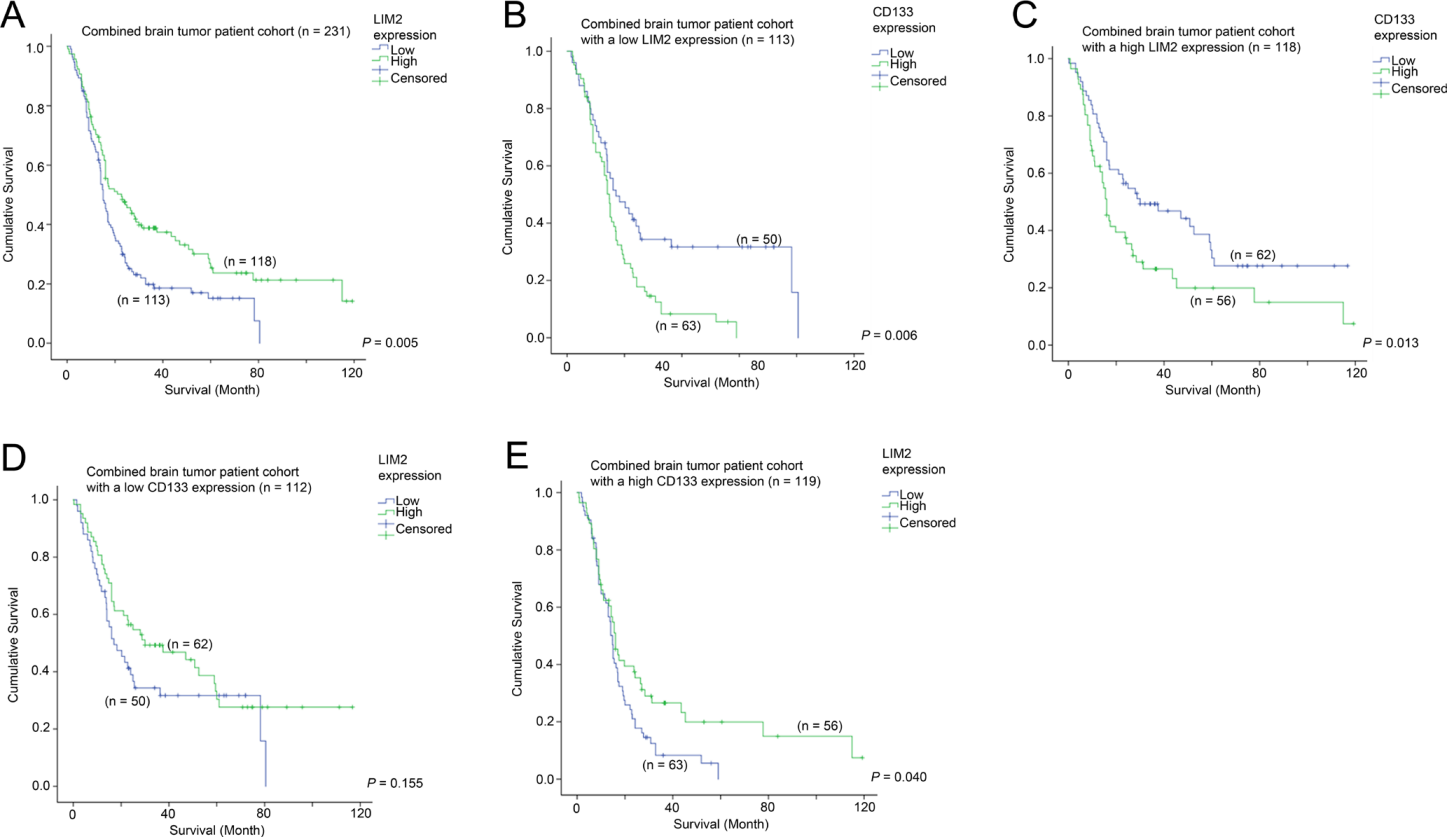
The association between LIM2 mRNA expression and survival (**A**) Kaplan-Meier analysis for LIM2 mRNA expression in the combined glioma patient cohort. Kaplan-Meier analyses for CD133 mRNA expression in patients with (**B**) a low level expression of LIM2 and (**C**) a high level expression of LIM2. Kaplan-Meier analyses for LIM2 mRNA expression in patients with (**D**) a low level expression of CD133 and (**E**) a high level expression of CD133.

Since a high level of CD133 was associated with a shorter survival in brain tumor patients, we sought to identify additional genes that could be used to further augment the predictive power of CD133, or to identify potential therapeutic targets for this group of patients with poor prognosis. By comparing CD133-high patients who were still alive and those who were deceased at data collection, we found that LIM2 expression was significantly differentially expressed. We went on to further investigate the prognostic significance of LIM2, and found that a low level of LIM2 was significantly correlated with a shorter survival time in patients with brain tumors (Figure 6A; *p* = 0.005). LIM2-low patients had a mean survival time of 25.3 months while LIM2-high patients had a mean survival time of 43.1 months. Expression of CD133 was significantly associated with survival in both LIM2-low (Figure 6B; *p* = 0.005) and LIM2-high (Figure 6C; *p* = 0.017) patients. However, in a different configuration, the association between LIM2 and survival was only observed in CD133-high patients (Figure 6E; *p* = 0.040), but not in CD133-low patients (Figure 6D; *p* = 0.155). Importantly, CD133 expression and LIM2 expression were independent prognostic markers in brain tumor patients as shown by multivariate Cox regression analysis (Table 4).

**Table 4:** Multivariate Cox-regression analysis for LIM2 and CD133

Factor	Hazard Ratio	95% Confidence Interval	*P*-value for inclusion
**CD133**	1.759	1.296–2.388	< 0.001
**LIM2**	0.679	0.502–0.919	0.012

### The association between CD133, VEGFa and survival

**Figure 7:**
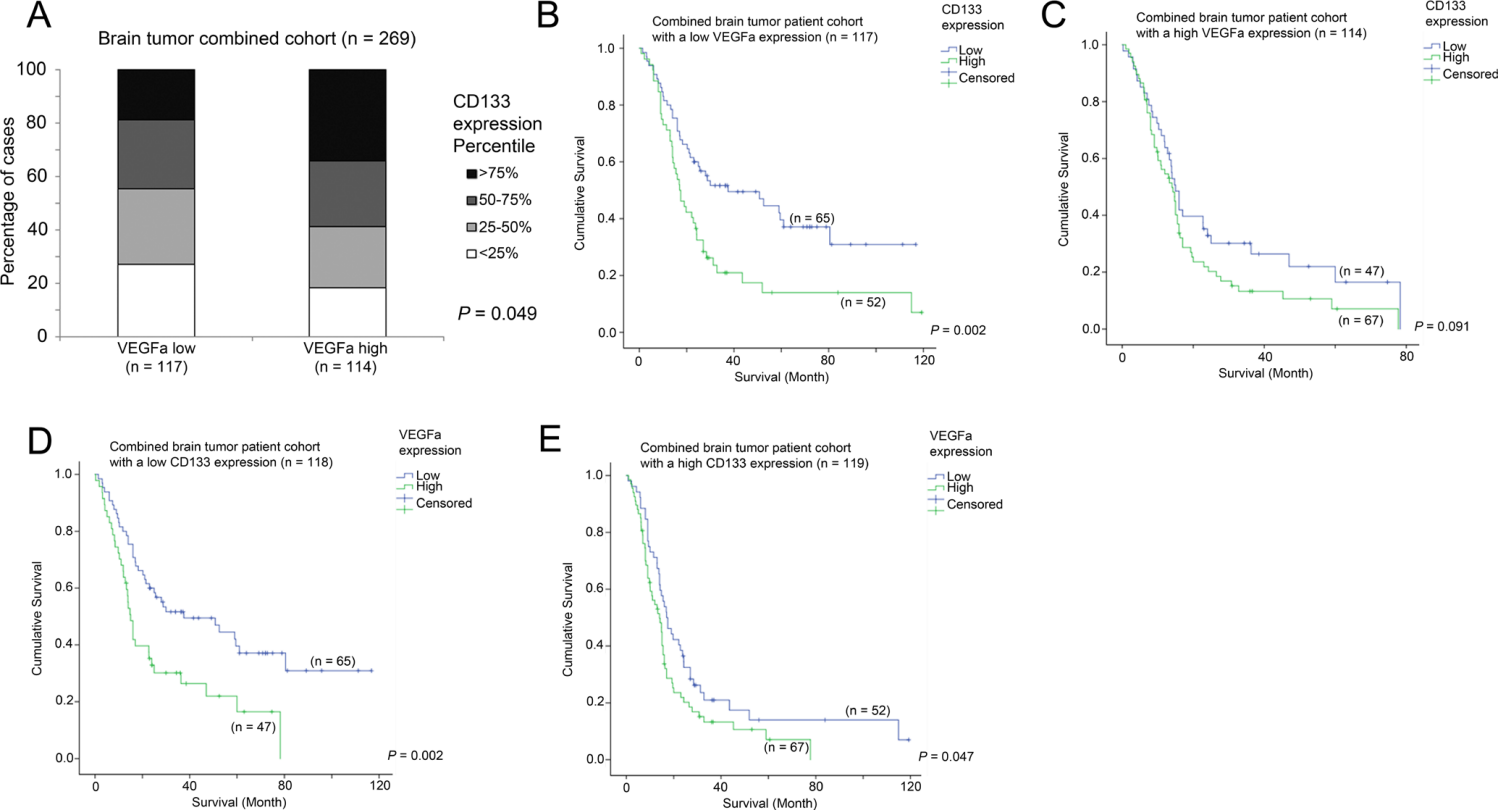
The association between VEGFa mRNA expression, CD133 mRNA expression and survival (**A**) A histogram showing the association between the expression levels of VEGFa and CD133. Kaplan-Meier analyses for CD133 mRNA expression in patients with (**B**) a low level expression of VEGFa and (**C**) a high level expression of VEGFa. Kaplan-Meier analyses for VEGFa mRNA expression in patients with (**D**) a low level expression of CD133 and (**E**) a high level expression of CD133.

We have previously shown that VEGFa is a prognostic marker in brain tumor patients [33]. In the present study, we found that CD133 expression was associated with survival. Interestingly, we further found that CD133 expression was significantly higher in VEGFa-high patients than in VEGFa-low patients (Figure 7A; *p* = 0.049), although CD133 was significantly associated with survival only in VEGFa-low patients (Figure 7B; *p* = 0.002) but not in VEGFa-high patients (Figure 7C; *p* = 0.091). On the other hand, VEGFa expression was significantly associated with survival in both CD133-low patients (Figure 7D; *p* = 0.002) and CD133-high patients (Figure 7E; *p* = 0.047). Importantly, multivariate Cox regression analysis showed that VEGFa expression and CD133 expression were independent prognostic markers in patients with brain tumors (Table 5).

**Table 5:** Multivariate Cox-regression analysis for VEGFa and CD133

Factor	Hazard Ratio	95% Confidence Interval	*P*-value for inclusion
**CD133**	1.611	1.197 – 2.170	0.002
**VEGFa**	1.692	1.257 – 2.277	0.001

### Identification of small molecules that could reverse the expression of CD133/HOX genes/LIM2-signature

Having shown that overexpression of CD133 and HOX genes and suppression of LIM2 were markers of poor prognosis, we used connectivity mapping to identify molecules that could reverse the expression of these genes. In the analysis, we found that the three chemotherapeutic agents most likely to reverse the gene expression profile identified were vinblastine (*p* < 0.001), vincristine (*p* < 0.001) and epirubicin (*p* < 0.001).

### The expression of CD133 and HOX genes in glioma cell lines

**Figure 8:**
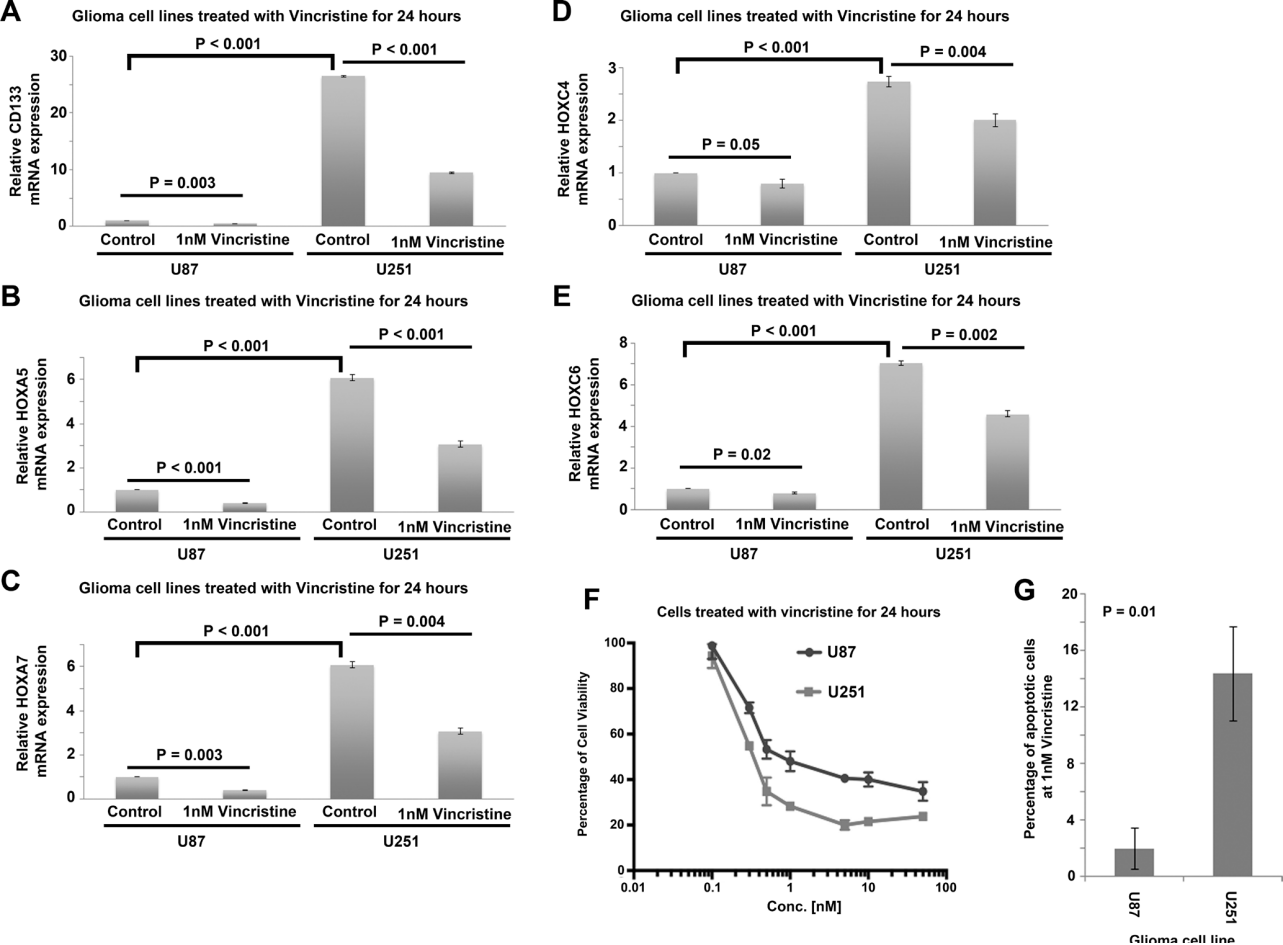
Expression of CD133 and HOX genes in human glioblastoma cell lines treated with vincristine (**A**) A histogram showing the relative expression of CD133 in control and vincristine treated U87 and U251 cells. (**B**) A histogram showing the relative expression of HOXA5 in control and vincristine treated U87 and U251 cells. (**C**) A histogram showing the relative expression of HOXA7 in control and vincristine treated U87 and U251 cells. (**D**) A histogram showing the relative expression of HOXC4 in control and vincristine treated U87 and U251 cells. (**E**) A histogram showing the relative expression of HOXC6 in control and vincristine treated U87 and U251 cells. (**F**) The percentage of cell viability of U87 and U251 cells treated with different concentration of vincristine determined by MTT assay. (**G**) The percentage of apoptotic cells in control and vincristine treated U87 and U251 cells as determined by Annexin V and PI staining through flow cytometry. Each data point with standard deviation represents three biological replicates.

mRNA expression of CD133 and HOX genes was tested in two glioma cell lines, U251 and U87. We found that CD133 (Figure 8A), HOXA5 (Figure 8B), HOXA7 (Figure 8C), HOXC4 (Figure 8D) and HOXC6 (Figure 8E) were significantly higher in U251 cells then in U87 cells (*P <* 0.001; Figure 8). This result supports our analysis in human glioma patient cohorts in which these genes are significantly correlated with each other. HOXA10 mRNA was not detected in U87 or in U251 cells. Importantly, we also confirmed the results of connectivity mapping, whereby treatment of U251 and U87 cells with vincristine for 24 hours downregulated the expression of CD133 (Figure 8A), HOXA5 (Figure 8B), HOXA7 (Figure 8C), HOXC4 (Figure 8D) and HOXC6 (Figure 8E). Furthermore, we found that U251 cells, which express higher level of CD133 and HOX genes were more sensitive to vincristine treatment than U87 cells in terms of cell viability (Figure 8F) and cell apoptosis (Figure 8G). These results suggest that CD133 and HOX genes can be downregulated by vincristine, and that a higher expression of these genes may be a predictive marker for vincristine sensitivity.

## DISCUSSION

In the present study, we demonstrated that CD133 overexpression predicts poor prognosis in patients with glioma. Importantly, for the first time, we showed that CD133 expression was highly correlated with expression of several HOX genes. These HOX genes are themselves prognostic markers in glioma, while the prognostic value for CD133 and HOX genes are mostly interdependent, with the exception of HOXA7. Moreover, we have identified that analysis of LIM2 expression could be used to further augment the prognostication of patients in the at-risk CD133-high subgroup. CD133 was also associated with VEGFa expression, suggesting a possible role of CD133 in promoting angiogenesis. Further analysis suggests that several chemotherapeutic agents may reverse the expression of CD133 and HOX genes.

An expression signature dominated by HOX genes and CD133 was a predictor for poor survival in glioma patients treated with concomitant chemoradiotherapy [34], suggesting that these genes may interact with one another. Multiple HOX genes have been shown to be overexpressed in glioma and confer prognostic value [30, 35], while HOXA10 has been shown to play a role in drug resistance and tumor recurrence [36]. In the present study, we found that HOXA5, HOXA7, HOXA10, HOXC4 and HOXC6 are prognostic markers in glioma patients. Interestingly, CD133 expression only confers prognostic value in patients expressing a low level of HOX genes, while expression of HOX genes confers prognostic value in patients expressing a low level of CD133, suggesting that the overexpression of either CD133 or HOX genes may be sufficient to drive glioma progression, while overexpression of both of CD133 and HOX genes may only slightly further enhance progression potential. Importantly, we found that CD133 and HOXA7 expressions were independent factors for predicting patient survival, suggesting that HOXA7 expression could add further value to the prognostic significance of CD133 expression. In view of this, CD133 or CD133 and HOX genes should be tested in clinical trial to evaluate whether the novel intervention could be effective specifically in this subgroup of glioma patients.

We have also identified LIM2 as a factor that could further predict survival in patients with CD133-high glioma. Little is known about the LIM2 gene. A comparative analysis of wild-type and LIM2-deficient mice revealed that LIM2 may have a role in maintaining cytoskeletal integrity, cell morphology and intercellular communication within lenses [29]. However, to the best of our knowledge, there seems to be no prior evidence implicating an involvement of LIM2 in cancer. On the other hand, there is evidence regarding the involvement of LIM2 in human brain development [37], so it is conceivable that dysregulation of LIM2 expression could manifest a malignant phenotype in brain cells. Further investigations are required to understand how LIM2 interacts with CD133 in glioma progression.

Several chemotherapeutic agents, namely vinblastine, vincristine, and epirubicin, were identified through connectivity mapping for their effect in reversing the CD133/HOX genes/LIM2 poor prognostic signature. Vinblastine has activity in pediatric low-grade gliomas [38]. However, its activity in adult human gliomas has not been studied. Vincristine has been shown to be effective in treating gliomas [39] and is one of the chemotherapeutic agents in a standard regimen (procarbazine, lomustine and vincristine) for the treatment of low-grade gliomas requiring postsurgical adjuvant chemotherapy [40]. In high-grade gliomas, vincristine in combination with procarbazine and lomustine was found to be comparable to temozolomide in terms of efficacy endpoints [41]. Since both vinblastine and vincristine are alkaloid-type chemotherapeutic agents that target microtubule dynamics through binding to tubulin, it is surprising that connectivity mapping suggested that they might regulate CD133, HOX genes and LIM2. Epirubicin acts through effects on DNA. It was encouraging that our *in vitro* analysis of cellular response to vincristine treatment supported our clinical cohort data, whereby the cells rich in the identified markers (U251) were significantly more susceptible to the cytotoxic properties of vincristine than the cells that expressed those markers at a relatively lower level (U87). It would be interesting to confirm this finding in clinical trials with CD133 as biomarker for patient stratification on which chemotherapeutic agents should be given.

Patients with glioma, in general, have poor prognosis compared to other types of solid tumors. In the present study, we have identified that a subgroup of patients with high level expression of CD133 and HOX genes had even worse prognosis than those with different expression patterns, suggesting the ineffectiveness of current treatment modalities to these patients. Whether reversing expression of these genes could be a novel therapeutic approach to prolong survival for this group of patients requires further investigation.

## MATERIALS AND METHODS

### Extraction of clinical and microarray gene expression data from glioma patient datasets

Three glioma patient datasets, GSE4271 [42], GSE4412 [43] and GSE7696 [34] were identified in the Gene Expression Omnibus (GEO) Database; datasets compiled using the HG-U133 microarray platform, and comprising > 70 patients for whom survival data were available, were included in this study. The patient characteristics for these three datasets are listed in Supplementary Table 1. Three other glioma patient datasets, GSE4290 [44], GSE16011 [45] and GSE52009, all of which comprise stage and expression data, were also included. Microarray gene expression data were retrieved from the data matrixes deposited to the GEO database by the original authors. R scripting was used to extract the expression values from probesets of CD133, HOXA5, HOXA7, HOXA10, HOXC4, HOXC6 and LIM2, and the clinical data from the data matrixes downloaded from GEO as previously described [46].

### Patient stratification

Expression levels were divided into four groups using quartile as the cut-off points. Expression levels were further divided into high and low levels using median expression level as the cut-off point for survival analysis.

### Correlations of gene expression levels and clinical data

All statistical analyses were performed using SPSS19.0. For Kaplan-Meier survival analysis, results were compared by log-rank test. Univariate Cox regression analysis was used to correlate the gene expression levels and patient survival, while multivariate Cox regression analysis was used to identify independent prognostic factors using a forward stepwise approach with a *p value* of 0.05 for inclusion. The three datasets with survival status available were combined into one to increase the sample size for detailed analysis. The associations between expression levels of genes were tested by Spearman's rank test. The association between tumor grade and gene expression was tested by Chi-Square test.

### Identification of genes co-expressed with CD133

The gene expression patterns of patients in the CD133-high and CD133-low subgroups were compared. Probesets that were differentially expressed between these two subgroups were identified by 2-sample Welch's *T-test*, avoiding the type I error due to unequal variances of the values of probesets between these two subgroups. Briefly, a Welch's t test was applied to each probeset corresponding to a particular gene in the data matrix using our own Java application, MyStats. *P* values and the differential expression in fold changes for all the probesets were generated as tab-delimited Excel worksheets for further analysis. Genes found to be co-expressed with CD133 were ranked by ascending *p*-values. The genes found to be co-expressed with CD133 that were common to all three patient datasets were analyzed further.

### Identification of gene target for patients with brain tumors overexpressing CD133

Patients who overexpressed CD133 were stratified into two groups based on mortality. Differential expressions of different probesets between patients in the CD133-high-alive subgroup and those in the CD133-high-deceased subgroup were identified as described above.

### Identification of potential inhibitory compounds targeting CD133/HOX genes/LIM2 glioma (Connectivity Map)

Gene expression connectivity mapping was performed using Statistically Significant Connection's Map (sscMap) to identify candidate small molecule compounds that may reverse the overexpression of CD133 and HOX genes and suppression of LIM2 gene [47–49]. The CD133/HOX genes/LIM2 probes were input to the Java application sscMap [49] as a query signature, and its association with the 6000 gene expression profiles generated by treating cancer cells with over 1000 small molecules were compared. The gene signature perturbation procedure, which increases the specificity of the output results, was applied as previously described [50]. All the small molecular compounds that were negatively associated with the input were sorted and ranked by their *p-value*, perturbation stability and standardized connection score. The *p-value* that was considered significant was set at a stringent threshold (*p* = 1/1309), ensuring that the results generated by sccMap would yield a maximum of one false positive small molecule over the 1309 small molecules tested in the sccMap [50].

### Cell culture

Two glioma cell lines, namely U87MG and U251 were used for the *in vitro* assays. U87MG and U251 were obtained from ATCC. They were cultured in Dulbecco's Modified Eagle's medium (Life Technologies) with 10% fetal bovine serum (FBS, Gibco, Life Technologies) and 1% penicillin/streptomycin (Gibco, Life Technologies). Cell cultures were maintained in a 37°C incubator with 5% CO2 and 95% air.

### RNA extraction and reverse transcription polymerase chain reaction (RT-PCR)

Cells were grown in a 6-well cell culture dish at a density of 2.5 × 10^6^ cells per well for 24 hour. RNA extraction was performed using RNeasy^®^ Mini Kit (QIAGEN) according to the manufacturer's instructions. Cells were disrupted by addition of lysis buffer, and the lysate was then put into a QIAshredder spin column for purification. RNA was eluted in the RNase-free water.

1 μg RNA was reverse transcribed into cDNA using High Capacity cDNA Reverse Transcription Kit (Thermo Fisher Scientific) according to the manufacturer's instructions. Each reverse transcription mix consisted of RT Buffer, dNTP Mix, RT Random Primers, MultiScribe^TM^ Reverse Transcriptase, nuclease-free H_2_O and RNA samples. RT-PCR was carried out in a C1000 Touch^TM^ Thermal Cycler (Bio-Rad) following the recommended thermal cycle program (25°C for 10 min, 37°C for 120 min, and 85°C for 5 min). cDNA products were stored in –80°C prior to use.

### Qualitative-PCR analysis of CD133 and HOX genes in glioma cell lines

QPCR reactions were carried out by mixing 5 μl TaqMan^®^ Universal PCR Master Mix (Thermo Fisher Scientific), 4 μl H2O and 1 μl cDNA sample. QPCR was conducted in a Mx3000P qPCR System (Agilent), with the following thermal cycling program: 95°C for 10 min, 40 cycles at 95°C for 15 sec and 60°C for 1 min. GAPDH was regarded as the reference gene, and relative abundance was calculated using the comparative Ct (2^−ΔΔCt^) method. Taqman qPCR probes were obtained from Thermo Fisher Scientific; CD133 (Hs01009257_m1), HOXA5 (Hs00430330_m1), HOXA7 (Hs00600844_m1), HOXA10 (Hs00172012_m1), HOXC4 (Hs00538088_m1) and HOXC6 (Hs00171690_m1); all probes spanned exons, preventing amplification of genomic DNA, and all probes had a PCR efficiency of 100% (± 10%) (https://www.thermofisher.com/hk/en/home/life-science/pcr/real-time-pcr/real-time-pcr-assays/why-choose-taqman-assays.html). The QPCR experiments were repeated three times for each batch of RNA, and three batches of RNA were extracted from three independent cell culture experiments.

### Cell viability and cell apoptosis upon Drug Treatment

Glioma cell lines were seeded at a density of 3000 per well in a 96-well plate (for MTT assay) and 3 × 10^5^ per well in a 6-well plate (for apoptosis assay) and were allowed to grow for 24 hours. Vincristine sulfate was added to the culture medium and the cells were harvested 24 hours post-treatment. Cell viability was measured by MTT assay, while cell apoptosis was measured by Alexa Fluor 488 Annexin V/Dead Cell Apoptosis Kit (Invitrogen) by flow cytometry.

## SUPPLEMENTARY MATERIALS FIGURES AND TABLES


