# Supplementary material for: CD133 in brain tumor: the prognostic factor
Source: Oncotarget. 2016 Dec 31;8(7):11144–59. doi: 10.18632/oncotarget.14406 (PMC5355253; doi:10.18632/oncotarget.14406)
Supplement: Supplementary file 1 [file oncotarget-08-11144-s001.pdf]

CD133 in brain tumor: the prognostic factor

Supplementary Materials

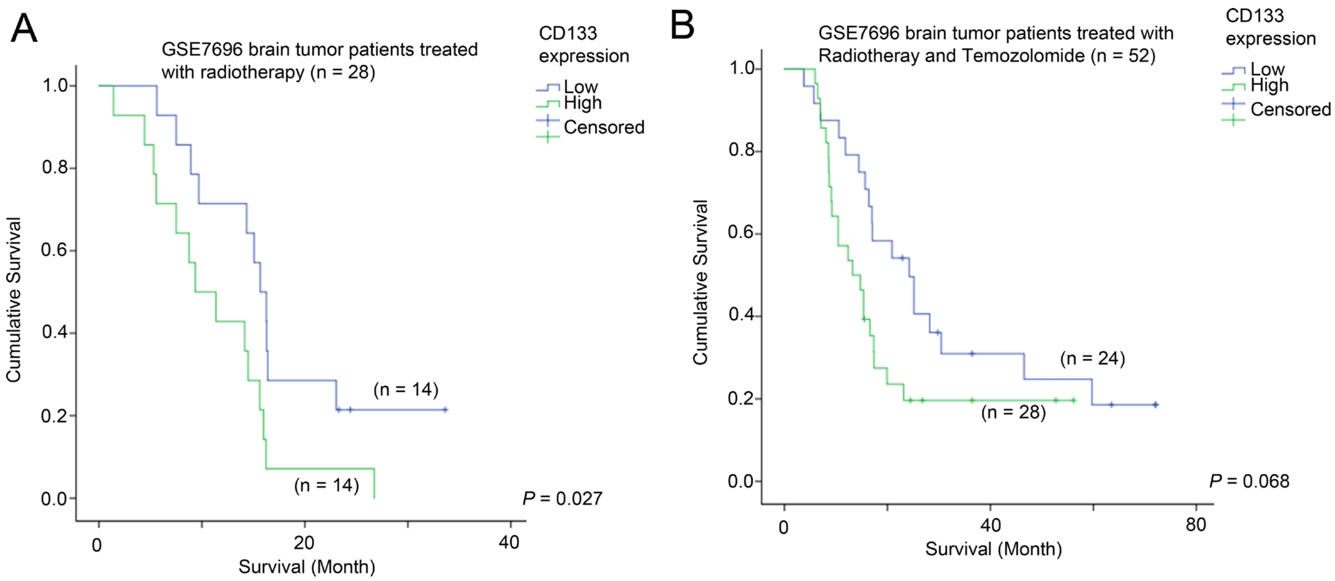

Supplementary Figure S1: The association between CD133 mRNA expression and survival in patients treated with radiotherapy alone or radiotherapy in combination with temozolomide in GSE7696 glioma patient cohort. (**A**) Kaplan-Meier analysis for CD133 mRNA expression in glioma patients treated with radiotherapy alone. (**B**) Kaplan-Meier analysis for CD133 mRNA expression in glioma patients treated with radiotherapy in combination with temozolomide.

Supplementary Table S1: Tumor grade and sex distribution in the 3 glioma datasets

GSE4271 (*n* = 100)	Number of cases	%
Tumor type		
Primary tumor	77	77.0
Recurrent (no survival data available)	23	23.0
Tumor grade (*n* = 77)		
Grade 1–3	21	27.3
Grade 4	56	72.7
Sex (*n* = 77)		
Female	25	32.5
Male	52	67.5
Censored patients	26	30.6
Follow up time	77	Median = 23.8 months<br>Range = 0.8–119.3 months
**GSE4412 (*n* = 74)**	**Number of cases**	**%**
Tumor grade		
Grade 1–3	24	32.4
Grade 4	50	67.6
Sex		
Female	46	62.2
Male	28	37.8
Censored patients	26	35.1
Follow up time	74	Median = 21.5 months<br>Range = 0.2–83.9 months
**GSE7696 (*n* = 80)**	**Number of cases**	**%**
Tumor grade		
Grade 1–3	0	0.0
Grade 4	80	80.0
Sex		
Female	21	26.3
Male	59	73.8
MGMT methylation		
Unmethylated	34	43.6
Methylated	44	56.4
Treatment		
Radiotherapy	28	45.0
Temozolomide/Radiotherapy	52	65.0
Censored patients	15	18.8
Follow up time	80	Median = 15.6 months<br>Range = 1.0–72.0 months

Supplementary Table S2: Mean survival time for patients with different expression characteristics in the combined glioma patient dataset

Expression characteristics	Mean survival time (95% CI) in month
CD133-high	25.7 (19.6–31.8)
CD133-low	45.7 (36.8–54.6)
HOXA5-high	26.3 (20.2–32.4)
HOXA5-low	44.9 (36.1–53.8)
HOXA7-high	23.7 (17.9–29.5)
HOXA7-low	47.2 (38.3–56.1)
HOXA10-high	24.1 (18.3–29.9)
HOXA10-low	46.1 (37.4–54.9)
HOXC4-high	30.4 (23.4–37.5)
HOXC4-low	41.0 (32.5–49.5)
HOXC6-high	27.1 (20.7–33.6)
HOXC6-low	44.1 (35.4–52.8)
LIM2-high	42.7 (34.3–51.2)
LIM2-low	25.5 (20.7–30.4)
CD133-high/HOXA5-high	22.8 (16.2–29.3)
CD133-high/HOXA5-low	30.3 (18.0–42.7)
CD133-low/HOXA5-high	33.1 (20.7–45.6)
CD133-low/HOXA5-low	50.6 (39.8–61.3)
CD133-high/HOXA7-high	20.7 (15.9–25.6)
CD133-high/HOXA7-low	32.4 (19.0–45.8)
CD133-low/HOXA7-high	29.4 (16.5–42.3)
CD133-low/HOXA7-low	53.0 (42.4–63.6)
CD133-high/HOXA10-high	19.9 (15.1–24.8)
CD133-high/HOXA10-low	32.7 (20.7–44.7)
CD133-low/HOXA10-high	32.2 (19.8–44.6)
CD133-low/HOXA10-low	52.2 (41.3–63.2)
CD133-high/HOXC4-high	25.7 (18.2–33.3)
CD133-high/HOXC4-low	27.6 (16.8–38.3)
CD133-low/HOXC4-high	40.5 (26.1–54.9)
CD133-low/HOXC4-low	47.4 (37.0–57.8)
CD133-high/HOXC6-high	23.4 (16.4–30.5)
CD133-high/HOXC6-low	29.0 (17.8–40.2)
CD133-low/HOXC6-high	33.4 (20.7–46.1)
CD133-low/HOXC6-low	51.0 (40.0–62.0)
CD133-high/LIM2-high	34.0 (22.7–45.4)
CD133-high/LIM2-low	17.8 (14.2–21.4)
CD133-low/LIM2-high	51.0 (39.1–62.9)
CD133-low/LIM2-low	34.4 (25.3–43.5)
CD133-high/VEGFa-high	19.7 (14.5–24.9)
CD133-high/VEGFa-low	32.0 (21.3–42.8)
CD133-low/VEGFa-high	27.9 (19.6–36.1)
CD133-low/VEGFa-low	56.1 (44.0–68.2)
